# Bilateral Staghorn Calculi With Stent Encrustation in a Patient With an Ileal Conduit Requiring Staged Endourological Procedures: A Case Report

**DOI:** 10.7759/cureus.116630

**Published:** 2026-09-21

**Authors:** Mohamed Ahmadi, Husain Jaafar, Ahmed A Al Rashed, Omran Hasan, Sayed Hasan Ebrahim, Mohamed Ali, Wafa Matar, Sara A Darwish, Abdolsalam Ahmadi

**Affiliations:** 1 Urology, Salmaniya Medical Complex, Manama, BHR; 2 Surgery, Urology Unit, Salmaniya Medical Complex, Manama, BHR

**Keywords:** dj stent, flexible ureteroscopy, ileal conduit, neurogenic urinary bladder, percutaneous nephrolithotomy (pcnl), staghorn stone, uretero-ileal anastomosis

## Abstract

An ileal conduit represents a surgical method of urinary diversion where the ureters are anastomosed to an isolated segment of the distal ileum, which is subsequently exteriorized as a stoma. Stones in ileal conduits are a recognized late complication of urinary diversion, arising from mucus production, urease-producing infection, and impaired drainage due to uretero-ileal stenosis.

We report a 30-year-old male with Down syndrome, neurogenic bladder, and chronic kidney disease who presented with bilateral staghorn calculi, stent encrustation, and a conduit calculus following prolonged loss to follow-up. He was managed with a staged, multimodal endoscopic approach combining percutaneous and transconduit techniques, achieving complete stone clearance with significant improvement in renal function. Such cases are technically challenging and require individualized management strategies, underscoring the importance of regular surveillance and early recognition of complications to prevent silent disease progression.

## Introduction

An ileal conduit represents a surgical method of urinary diversion where the ureters are anastomosed to an isolated segment of the distal ileum, which is subsequently exteriorized as a stoma [1]. This procedure is most commonly performed following radical cystectomy for bladder cancer [1]. In cases of neurogenic bladder, the procedure is generally reserved as a last resort, especially when less invasive management options have proven ineffective or unsuitable, contributing to diversion rates of approximately 68% in Zillioux et al.'s cohort [2]. The primary objectives of this intervention are to achieve low-pressure urinary storage and effective bladder emptying while safeguarding the upper urinary tract [2].

Complications related to ileal conduit surgery are classified into early and late events. Late complications may include parastomal hernia, ureteroileal stenosis, and hyperchloremic metabolic acidosis [3].

The use of intestinal tissue in urinary diversion inherently increases the risk of stone formation, a long-term complication occurring in about 3.5% of cases [4]. The ileal segment continuously secretes mucus, which can serve as a nidus for stone development and obstruction [5]. Persistent bacteriuria, although often asymptomatic, also predisposes to stone formation, particularly with urease-producing bacteria such as *Proteus mirabilis*, which promote the formation of struvite stones [5].

Managing calculi in patients with ileal conduits presents unique clinical and technical challenges owing to altered anatomy and the biological properties of the intestinal segment used in diversion. Unlike a normal urinary system, where retrograde access is facilitated through the bladder and natural ureteral orifices, the ureteroileal anastomoses create a more complex landscape that complicates endoscopic approaches [6].

Additionally, ureteroileal stenosis is a common late complication that may hinder the passage of ureteroscopes or stents. Spontaneous stone passage, typically seen in normal anatomy, is less likely in these cases due to impaired drainage and the absence of natural peristaltic activity. Similar difficulties are encountered in the management of stones within horseshoe kidneys or calyceal diverticula, where gravity and narrow openings delay fragment clearance after procedures such as extracorporeal shockwave lithotripsy (ESWL) [6].

While less frequently observed than in patients with neobladders, ileal conduits can also contribute to hyperchloremic metabolic acidosis. This occurs because the bowel segment absorbs acid and chloride from the urine, altering the urinary environment and potentially facilitating stone formation [5].

Given these challenges, percutaneous nephrolithotomy (PCNL) is often preferred for the removal of larger renal calculi in this patient population, providing direct access to the kidney, enabling complete stone clearance, and avoiding the complications associated with conduit-based interventions [7].

In this report, we present a rare case of a patient with Down syndrome and a long-standing ileal conduit who failed to follow up regularly. The patient developed ureteroileal stenosis with stent encrustation and bilateral staghorn calculi and was successfully managed with a staged endoscopic approach. This case emphasizes that the management of stone disease in patients with ileal conduits should be tailored individually, considering factors such as stone location, anastomotic anatomy, and patient fitness. Due to the rarity of such cases, we believe this report contributes valuable insights to the limited existing literature and underscores the importance of regular surveillance to prevent silent disease progression in this population.

## Case presentation

A 30-year-old male patient known to have Down syndrome, hypothyroidism, chronic kidney disease, and neurogenic bladder presented to the Emergency Department with a five-day history of nonspecific abdominal and right flank pain, accompanied by nausea, vomiting, and lethargy.

Detailed history revealed that the patient had undergone cystectomy and ileal conduit construction in 2015 due to neurogenic bladder complications. Since then, he had experienced multiple episodes of urinary tract infections (UTIs), predominantly caused by ESBL-producing *Escherichia coli*, necessitating repeated hospital admissions. He also developed ureteroileal anastomotic stenosis, requiring multiple nephrostomy placements and double J (DJ) stent insertions and exchanges. He was also lost to follow-up, and the last DJ exchange was conducted three years ago.

On examination, the patient was alert and hemodynamically stable. Abdominal examination was unremarkable, except for signs of inflammation over the ileal conduit, including erythema and warmth. There was no costovertebral angle tenderness, and genital examination revealed an undescended left testis.

Initial laboratory investigations (Table 1) indicated acute-on-chronic kidney injury, UTI, and elevated inflammatory markers. Urinalysis and urine culture yielded *P. mirabilis*. Ultrasonography demonstrated significant right hydronephrosis with cortical thinning, suggestive of chronic obstruction, and possible stent encrustation. Additionally, a large left pelvic calculus measuring approximately 3.5 cm was noted, with moderate hydronephrosis and properly positioned DJ stents.

**Table 1:** Results of lab values upon patient's initial presentation

Test description	Lab value	Unit	Reference range
WBC	7.9	10^9/L	3.5 - 10.5
Hb	108	g/L	135 - 175
Platelets	329	10^9/L	150 - 450
Createnine	4.9	mg/dL	0.7 - 1.2
Urea	219	mg/dL	5 - 50
Chloride	115.8	mmol/L	98 - 107
CRP	90	mg/L	< 10 mg/L
Na	130	mmol/L	135 - 145
K+	5	mmol/L	3.5 - 5.1
HCO3-	15	mmol/L	22 - 29

Further imaging with computed tomography (CT) provided a detailed assessment (Figures 1-2), revealing hyperdense material within the ileal conduit surrounding the stent, consistent with encrustation or stone fragments measuring approximately 21 × 12 mm. The right kidney showed severe hydronephrosis and hydroureter with pelvicalyceal ballooning. The proximal ends of the DJ stents were encased in hyperdense material (2.8 × 1.5 cm), with similar findings in the left kidney, including extensive hydronephrosis, hydroureter, and surrounding stone encrustation. Bilateral ureteral thickening and periureteral fat stranding were also observed.

**Figure 1:**
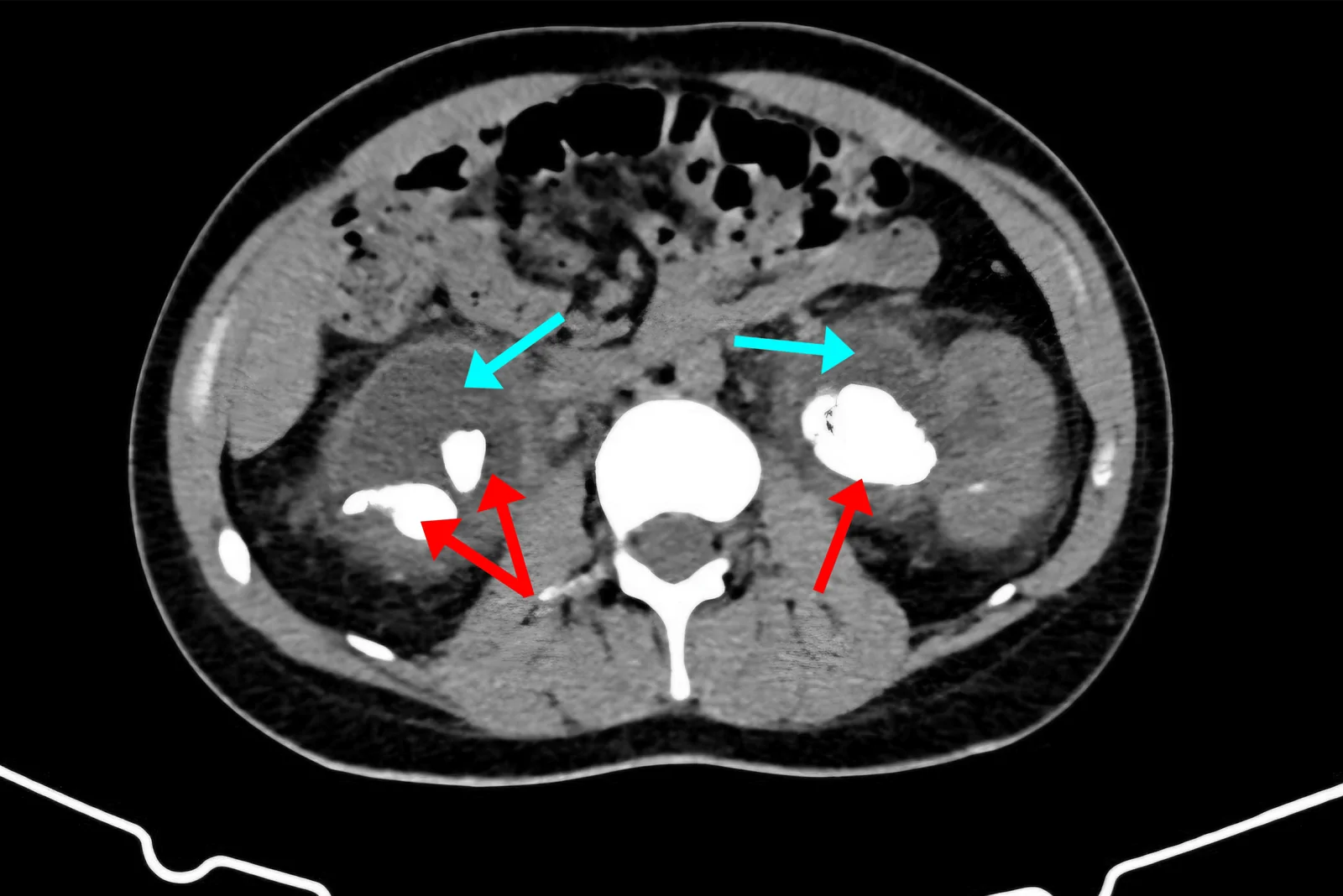
Axial CT KUB view Red arrows show bilateral large staghorn stones. Blue arrows show bilateral severe hydroureteronephrosis.

**Figure 2:**
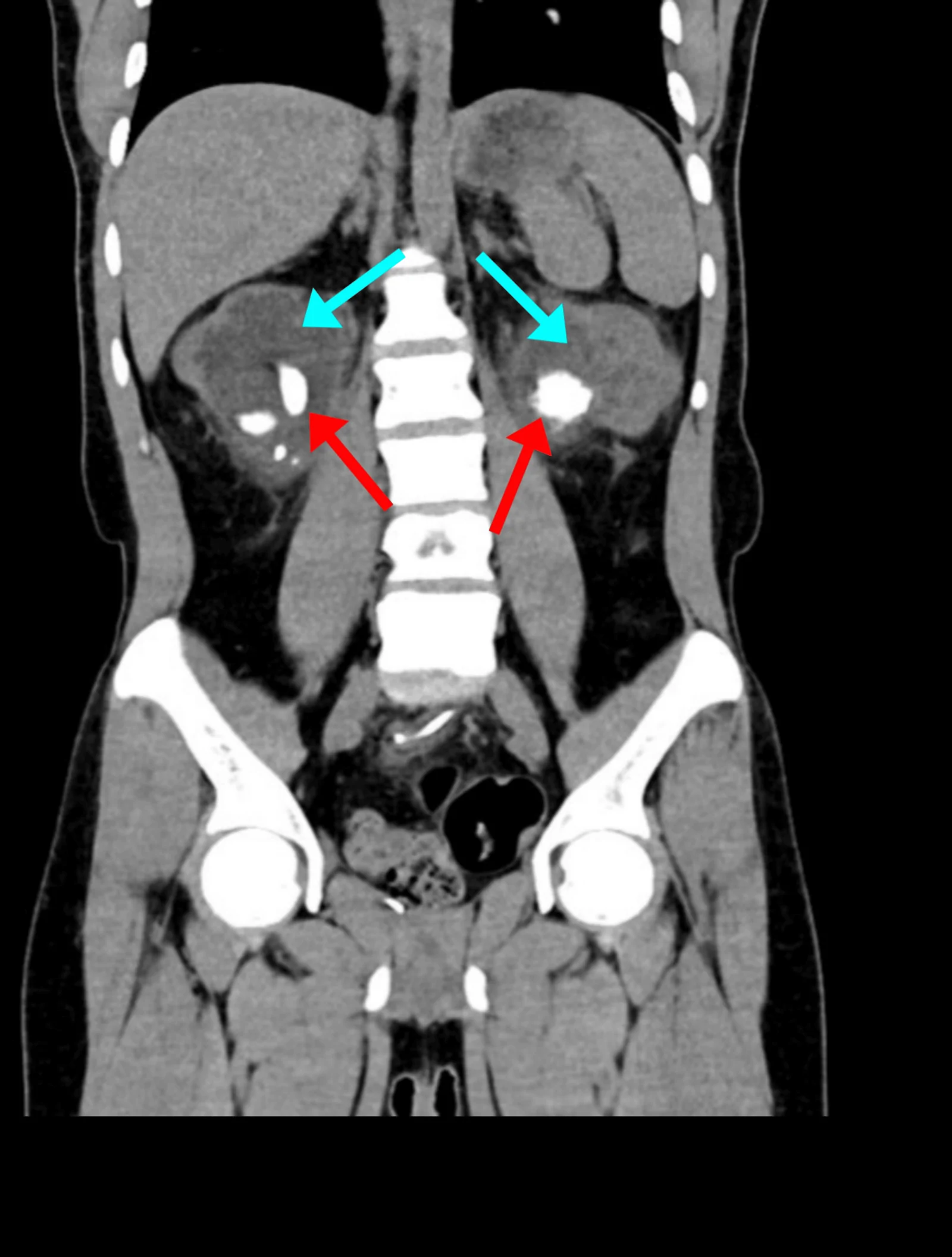
Coronal CT KUB view Red arrows show bilateral large staghorn stones. Blue arrows show bilateral severe hydroureteronephrosis.

Initial management involved bilateral nephrostomy placement for urinary diversion, combined with broad-spectrum intravenous antibiotics tailored to culture sensitivity and a new urine culture taken directly from the nephrostomy, which again revealed the same organism.

After resuscitation, the patient’s family was counseled regarding the need for definitive management of the stone burden and stent encrustation. A CT urogram with contrast was performed (Figures 3-5), confirming proper positioning of bilateral nephrostomy tubes, with ongoing decompression of the prior hydronephrosis and persistent stone and encrustation burden. Filling defects along both DJ stents and within the conduit were visualized, though antegrade contrast studies confirmed ureteral patency and absence of leaks.

**Figure 3:**
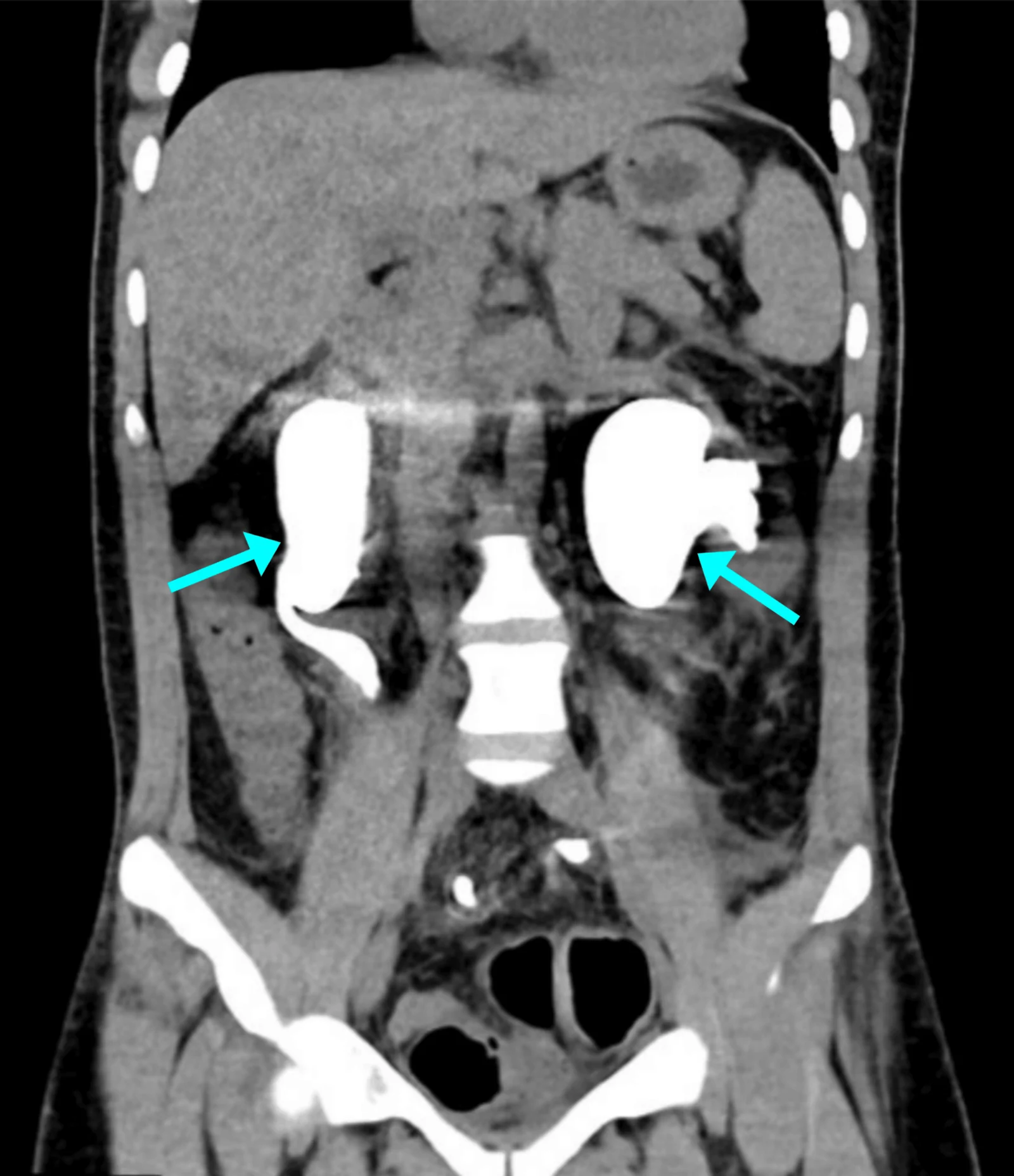
Coronal view of CT urogram with IV contrast Blue arrows show bilateral hydroureteronephrosis.

**Figure 4:**
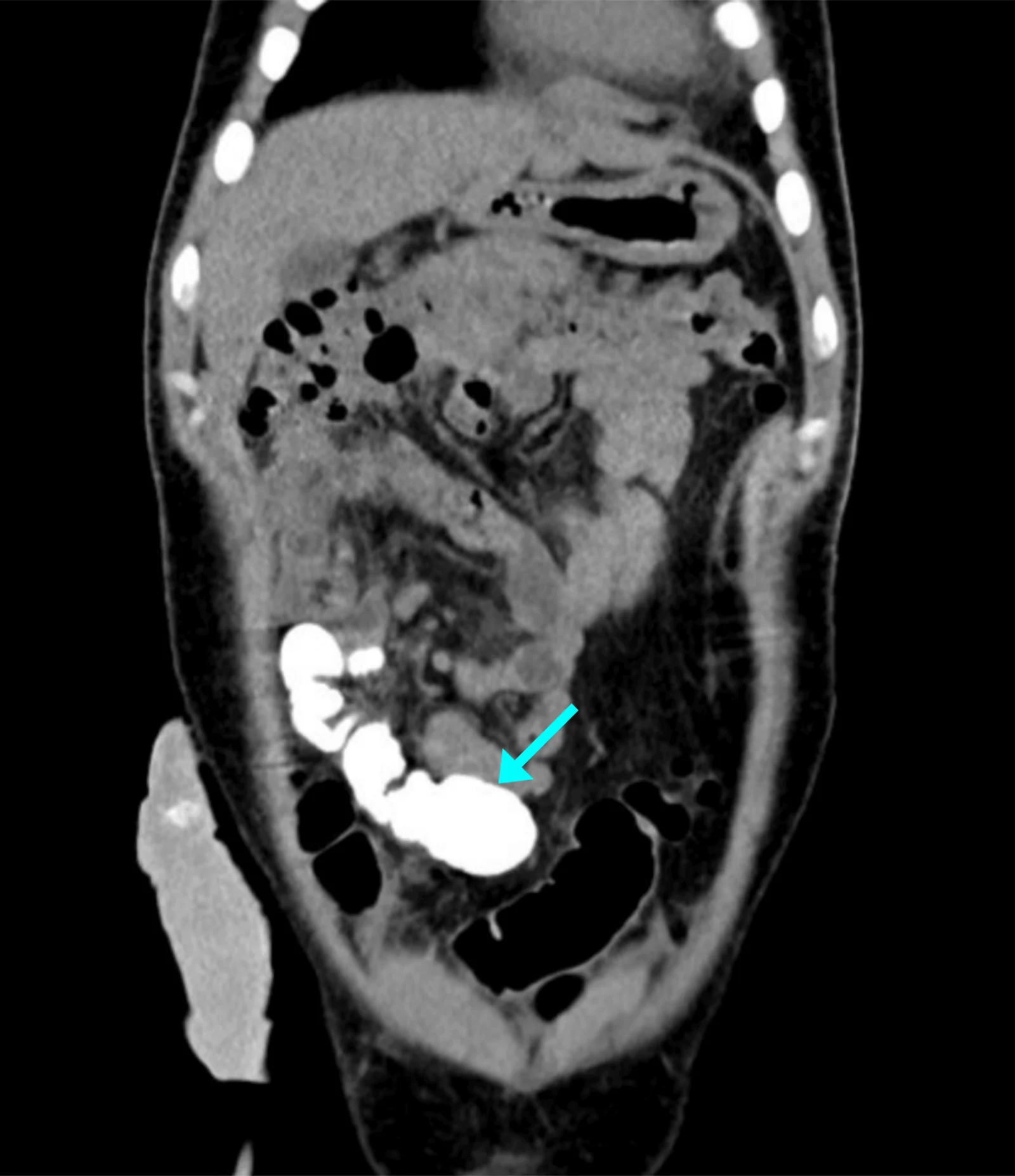
Coronal view of CT urogram with IV contrast Blue arrow shows contrast reaching the ileal conduit pouch, confirming ureteral patency.

**Figure 5:**
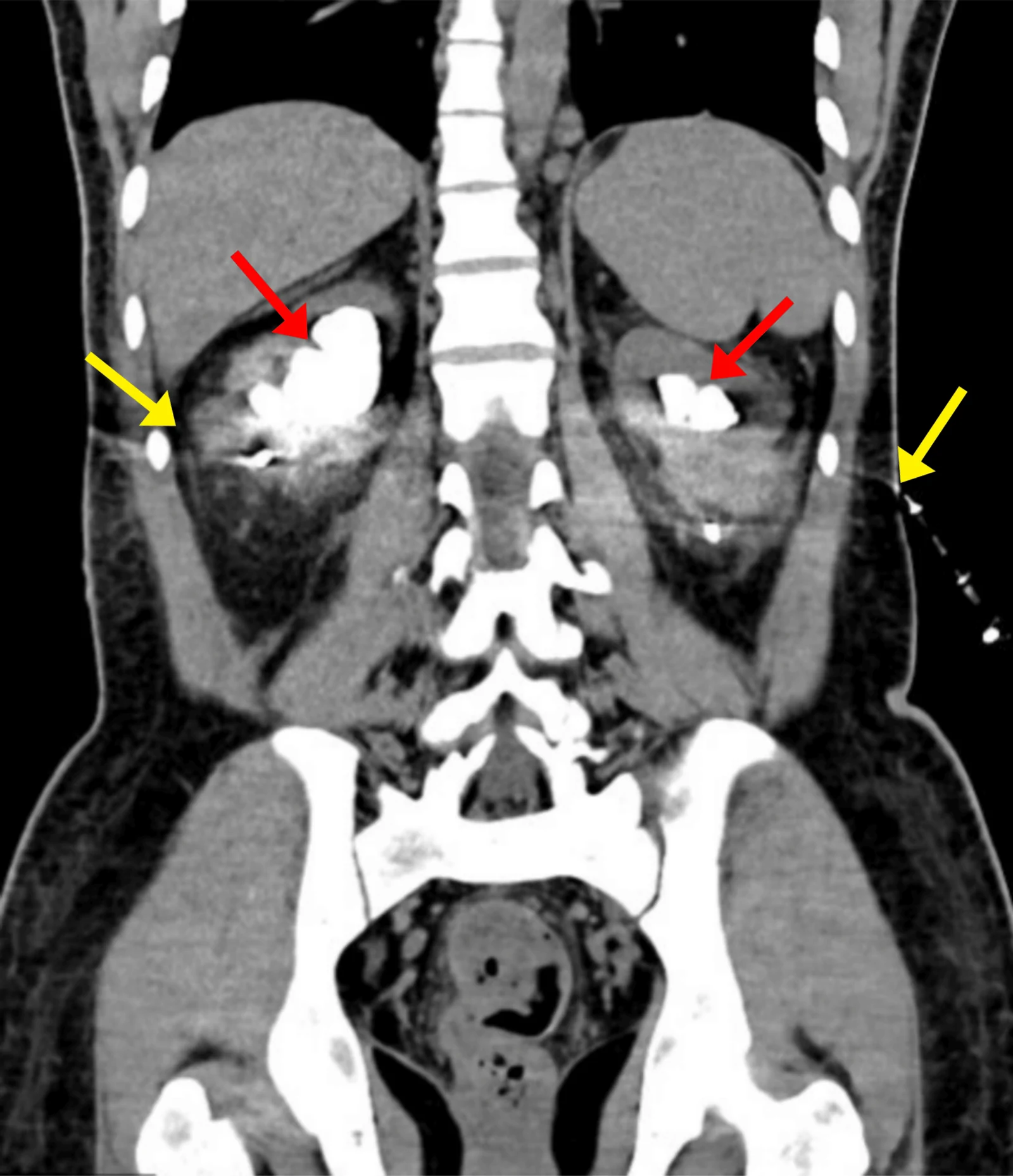
CT urogram coronal view Red arrows show bilateral staghorn stones. The proper positioning of bilateral nephrostomies is indicated by yellow arrows.

Surgical intervention was discussed with the family and initially planned in five stages: lithotripsy of pouch stones, left PCNL, left flexible ureteroscopy and DJ exchange, right PCNL, and finally, right flexible ureteroscopy and DJ exchange. The stages of surgical intervention were completed within two surgical sessions.

The initial session involved flexible cystoscopy of the ileal conduit, followed by laser lithotripsy and complete clearance of the conduit stones. The patient was positioned supine and prepped and draped in standard fashion for lower urinary tract access. A 15.5-Fr flexible cystoscope (Pusen; Zhuhai Pusen Medical Technology Co., Ltd., Zhuhai, China) was introduced per conduit under direct vision into the conduit, where bilateral DJ stents were visualized in situ along with the conduit calculi. A Holmium laser fiber (325 µm; Karl Storz ENDOSKOPE laser; KARL STORZ SE & Co. KG, Tuttlingen, Germany) was passed through the working channel, and laser lithotripsy was performed, dusting the stones into pieces small enough for clearance with irrigation and suction. Hemostasis and stent positions were confirmed. A 16-Fr Foley catheter was introduced through the pouch opening and fixed as the patient was repositioned for the left PCNL.

The patient was then repositioned to the prone position, and the left flank and retroperitoneum were prepped and draped. Antegrade pyelography was performed via the existing left nephrostomy to opacify the collecting system under fluoroscopic guidance. Using the C-arm, the target calyx was identified, and percutaneous puncture was performed using the triangulation technique with an 18G PCNL needle, confirmed on orthogonal fluoroscopic views. A 0.035-inch stiff guidewire was passed through the needle into the collecting system, with fluoroscopic confirmation of coiling within the renal pelvis. Tract dilatation was then performed over the guidewire under fluoroscopic control, and an Amplatz 16-Fr working sheath was placed to establish the nephrostomy tract. A nephroscope was introduced through the sheath, and the Swiss Lithoclast TRILOGY system was used to fragment and simultaneously aspirate the renal stones on the left side, with stone clearance confirmed under direct vision and fluoroscopy. A flexible ureteroscope (Karl-Storz) was then passed to inspect the ureter in antegrade fashion; the existing left DJ stent was localized, freed of adherent stone fragments using a holmium laser (KARL STORZ ENDOSKOPE laser), and removed. A new 4.7-Fr, 24-cm DJ stent was placed under fluoroscopic guidance, with the proximal coil confirmed in the renal pelvis and the distal coil in the conduit. A 16-Fr Malecot nephrostomy tube was left in situ, hemostasis was confirmed, and the procedure was concluded. The patient was shifted to recovery in stable condition, and the nephrostomy tube was removed on postoperative day (POD) 2.

Ten days later, the patient was brought back for a right PCNL following the same steps as on the left side. The patient was positioned prone, and the right flank was prepped and draped. Antegrade pyelography was performed via the existing nephrostomy under fluoroscopic guidance, followed by calyceal puncture using the triangulation technique. A guidewire was placed, tract dilatation was performed, and an Amplatz sheath was inserted, similar to the first session on the left side. A nephroscope was introduced, and the Swiss Lithoclast TRILOGY system was used to fragment and clear all stones on the right side, with clearance confirmed both endoscopically and fluoroscopically. The right DJ stent status was addressed accordingly. A 16-Fr Malecot nephrostomy tube was placed, and the procedure was concluded with the patient transferred to recovery in stable condition. The nephrostomy tube was removed on POD 2.

Postoperatively, the patient’s renal function improved significantly (Figures 6-7), with clearance of the UTI. The results of the stone analysis indicated the presence of struvite stones, also known as triple-phosphate stones (comprising calcium, ammonium, magnesium, and phosphate). These findings are consistent with recurrent UTIs and staghorn calculi. He was closely monitored, stabilized, and eventually discharged in good condition.

**Figure 6:**
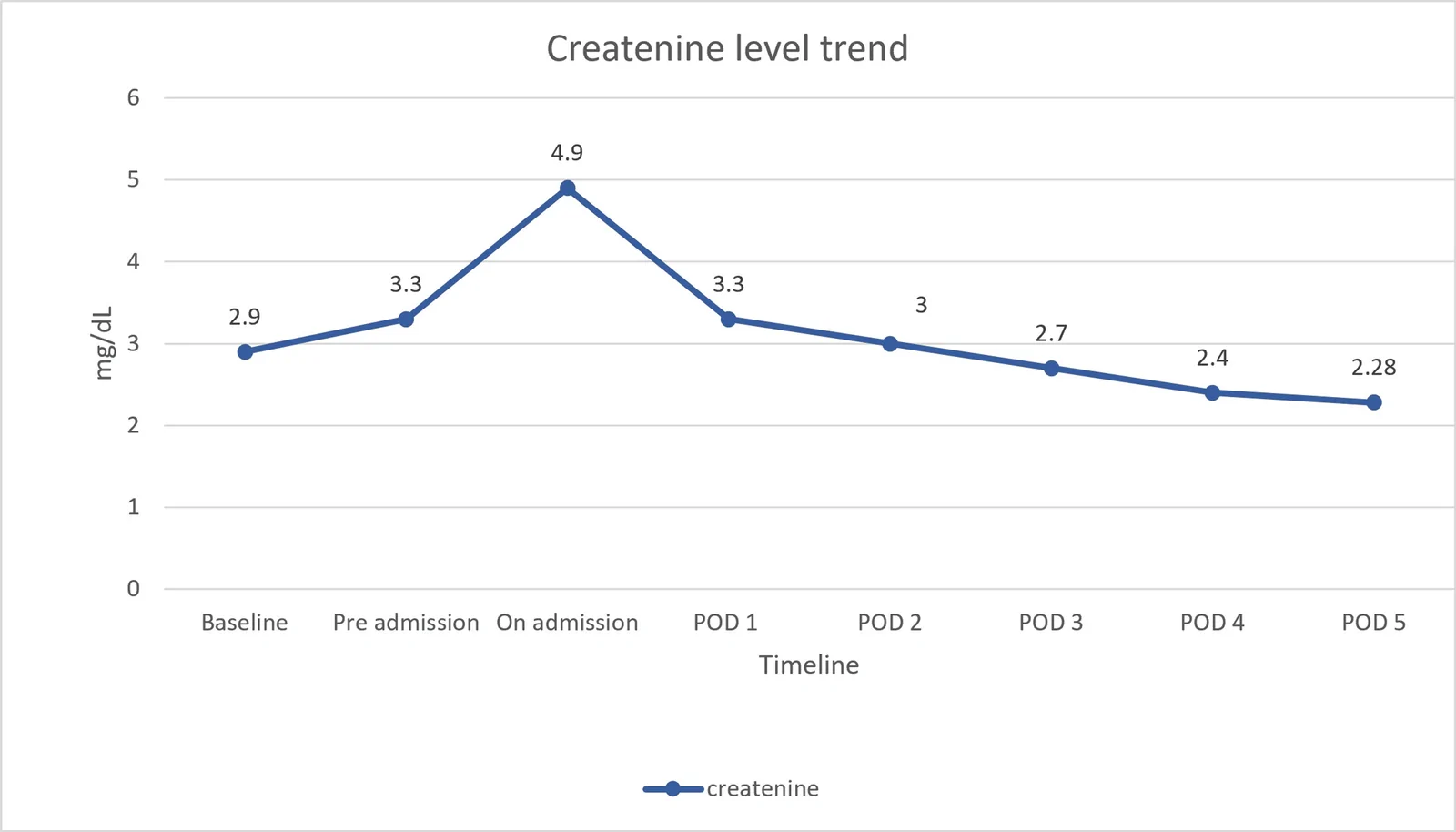
Creatinine level trend

**Figure 7:**
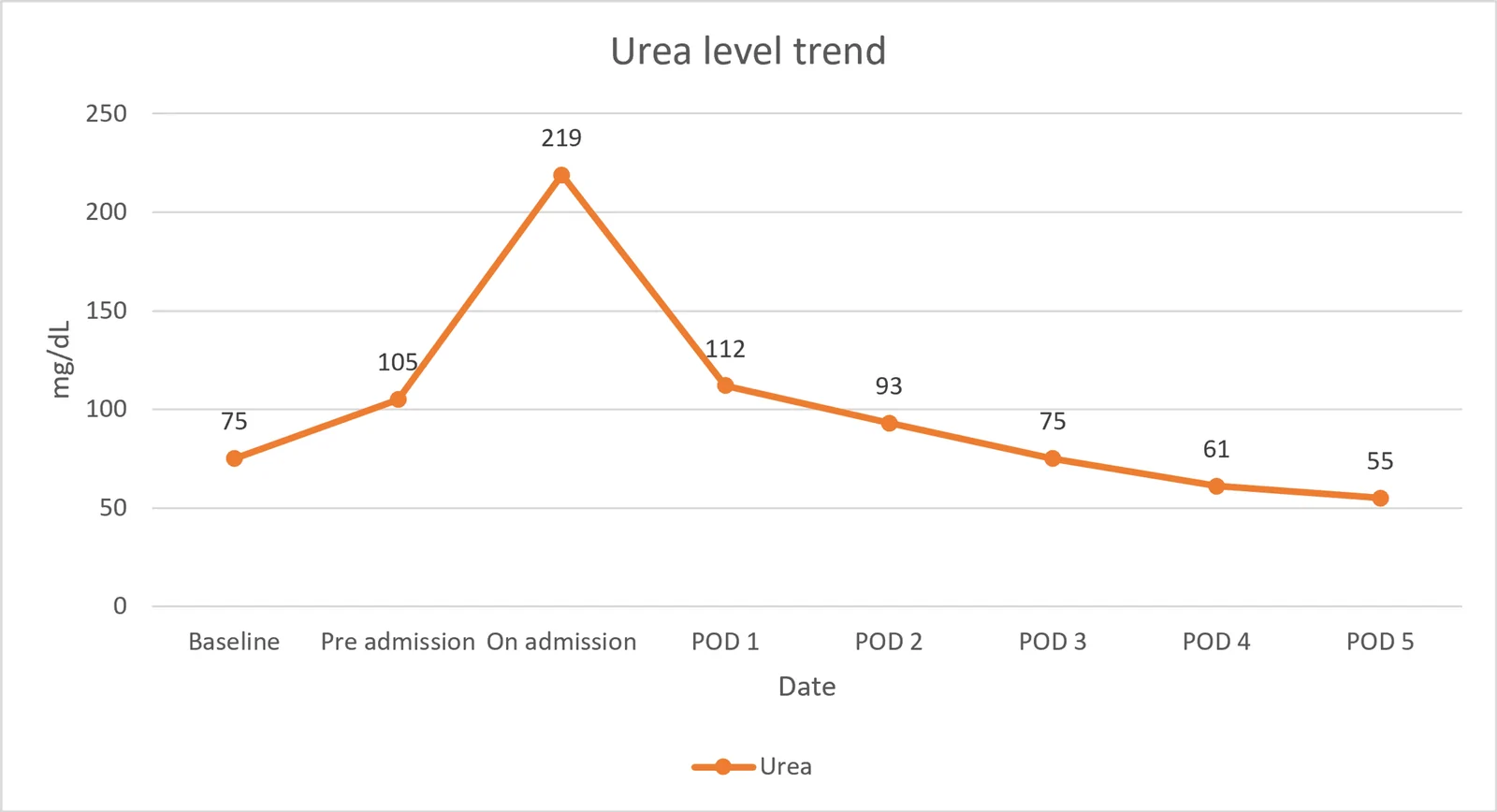
Urea level trend

Family counseling included discussions regarding further management, including regular DJ stent exchanges versus revision of the conduit. 

## Discussion

This case illustrates several challenges unique to stone management in patients with long-standing ileal conduits, particularly in the context of neurogenic bladder and loss to follow-up. The patient's stone burden, bilateral staghorn-type calculi, stent encrustation, and pouch calculi developed against a background of recurrent *P. mirabilis* bacteriuria, ureteroileal stenosis, and a three-year gap since his last DJ stent exchange, underscoring how interruption in routine surveillance can allow a manageable complication to progress to a bilateral, near-emergent stone burden with acute-on-chronic renal impairment.

The pathophysiology in this patient reflects the convergence of several known risk factors described in the literature. Chronic mucus production by the ileal segment provides a nidus for crystallization, while persistent infection with a urease-producing organism such as *P. mirabilis* drives struvite stone formation through urease-mediated urea hydrolysis and urinary alkalinization [5]. Superimposed ureteroileal stenosis, a recognized late complication of ileal conduit surgery, further impairs drainage and clearance of debris, creating a self-perpetuating cycle of stasis, infection, and stone growth [3,6]. This patient's prolonged interval without stent exchange likely allowed encrustation to progress along both stents and within the conduit itself, compounding the obstructive burden bilaterally. This mechanism mirrors that described by Katsui and Arakawa [8], whose patient's staghorn calculus proved on analysis to be composed predominantly of magnesium ammonium phosphate and calcium phosphate, the classic signature of infection-related stone formation in a diverted urinary tract, closely paralleling the struvite-favoring picture driven by *P. mirabilis* in our patient.

Endourologic management in diverted urinary tracts is technically more demanding than in patients with intact native anatomy. The absence of a bladder and native ureteral orifices eliminates the option of straightforward retrograde ureteroscopic access, and ureteroileal anastomotic stenosis, as seen in this patient, can further obstruct scope passage [6]. These anatomical constraints, combined with the reduced likelihood of spontaneous fragment passage due to the nonperistaltic, absorptive nature of the ileal segment, mean that percutaneous access is often favored over purely retrograde approaches for larger renal calculi, consistent with prior reports supporting PCNL as the preferred modality in this population [7].

Furthermore, Katsui and Arakawa [8] offer a useful counterpoint by illustrating a purely retrograde approach, achieved via staged flexible ureteroscopy through the conduit itself, for a large right renal staghorn calculus. Their case demonstrates that retrograde feasibility in ileal conduit patients depends heavily on the configuration of the ureteroileal anastomosis: a Wallace-type anastomosis, in which both ureters are joined into a common plate, provided a wide, stable entry point that tolerated repeated ureteroscope passage and, in later sessions, safe insertion of a ureteral access sheath. They contrast this with Nesbit-type anastomoses, where each ureter is implanted separately and orifices tend to be narrower and more angulated, limiting maneuverability and precluding sheath use. This anatomical dependency is directly relevant to our patient, whose known ureteroileal anastomotic stenosis and history of multiple stent exchanges suggest an unfavorable anastomotic configuration that would likely have limited pure retrograde access, reinforcing our decision to rely on an antegrade percutaneous strategy for the renal components of the stone burden rather than attempting staged retrograde ureteroscopy as Katsui and Arakawa did [8].

Comparison with recently reported alternative strategies helps place our approach in context. Eslahi et al. [9] described an 85-year-old man with a distal ureteral stone and lower-pole renal stones managed via a combined antegrade technique: a percutaneous nephrostomy tract was used to introduce a semirigid ureteroscope antegrade down to the distal ureter, with holmium laser lithotripsy used to fragment the impacted stone and irrigation used to flush fragments into the ileal conduit for clearance. That report highlights antegrade ureteroscopy as a reproducible, minimally invasive option specifically for distal ureteral stones close to the ileal conduit orifice, a location that is often difficult to reach either by pure percutaneous puncture or by retrograde means because of anastomotic narrowing. In our patient, distal ureteral and stent-related debris within the conduit itself was instead addressed by direct transconduit flexible cystoscopy with laser lithotripsy, which achieved a comparable goal (clearance of stone material adjacent to the anastomosis) without requiring a dedicated antegrade ureteroscopic pass down the full length of the ureter.

Taken together, these cases illustrate that no single endoscopic strategy fits all ileal conduit patients with urolithiasis and that technique selection should be individualized based on stone location, anastomotic anatomy, and the practical accessibility of the conduit itself. Distal ureteral or peri-anastomotic stones may be well suited to antegrade ureteroscopy via an existing nephrostomy tract, as shown by Eslahi et al. [9], particularly when the stone lies close to the ileal conduit orifice, while renal stones with favorable Wallace-type anastomotic anatomy may be amenable to staged retrograde flexible ureteroscopy through the conduit, as shown by Katsui and Arakawa [8], avoiding the morbidity of percutaneous tracts altogether. Large, bilateral stone burdens with known anastomotic stenosis and encrustation, as in our patient, may instead be better served by a hybrid approach combining bilateral PCNL for renal clearance with direct transconduit flexible cystoscopy and laser lithotripsy for pouch and peri-stent stone material, allowing the entire disease burden to be addressed in a minimum number of anesthetic sessions.

In this case, a staged, combined-access approach was used: antegrade access via the existing nephrostomy tracts guided percutaneous puncture and tract dilatation for bilateral PCNL, while a flexible cystoscope introduced through the conduit itself allowed direct visualization and laser lithotripsy of the pouch calculi and stented segments. This hybrid antegrade-transconduit strategy allowed complete stone clearance to be achieved in two surgical sessions rather than the five originally planned, reducing the number of anesthetic exposures in a patient with multiple comorbidities, including Down syndrome and chronic kidney disease. The use of antegrade pyelography via existing nephrostomy tracts to opacify the collecting system rather than attempting fresh retrograde ureteral catheterization through the diverted tract was a practical adaptation that simplified calyceal targeting under fluoroscopy, an approach conceptually similar to the antegrade pyelography used by Katsui et al. [8] for their distal ureteral stone.

The patient's renal function improved substantially following decompression and stone clearance, reflecting the reversible component of his acute-on-chronic kidney injury that was attributable to obstruction and infection rather than to fixed structural damage alone. This recovery reinforces the importance of prompt intervention once significant stone burden and encrustation are identified rather than pursuing prolonged conservative management with nephrostomy drainage alone.

This case also highlights the importance of structured long-term surveillance in patients with ileal conduits, particularly those with neurogenic bladder who may have limited capacity for self-advocacy or symptom reporting, as was the case here given the patient's Down syndrome. Regular stent exchange schedules, periodic imaging, and monitoring of renal function and urine cultures are essential to prevent the silent progression of encrustation and stone formation seen in this patient. Both Katsui and Arakawa [8] and Eslahi et al. [9] likewise emphasize that technique selection and long-term outcomes in this population hinge on individualized assessment of anatomy and disease burden rather than a one-size-fits-all algorithm. Following resolution of the acute presentation, the family was counseled regarding the relative merits of continued regular DJ stent exchange versus conduit revision, a decision that should weigh the patient's overall functional status, anesthetic risk, and capacity for sustained follow-up.

## Conclusions

Stones in ileal conduits arise from mucus production, urease-producing infection, and impaired drainage from ureteroileal stenosis, and their management must be individualized. This case shows that a hybrid antegrade-transconduit strategy, bilateral PCNL combined with transconduit flexible cystoscopy and laser lithotripsy, can achieve complete clearance in a complex patient with minimal anesthetic exposure.

Stone location, anastomotic anatomy, and patient fitness should guide technique selection, while regular surveillance and timely stent exchange remain essential to prevent silent disease progression.
